# Csnk1a1 inhibition modulates the inflammatory secretome and enhances response to radiotherapy in glioma

**DOI:** 10.1111/jcmm.16767

**Published:** 2021-07-03

**Authors:** Guanzheng Liu, Huan Li, Wanhong Zhang, Jiefeng Yu, Xu Zhang, Runqiu Wu, Mingshan Niu, Xuejiao Liu, Rutong Yu

**Affiliations:** ^1^ Insititute of Nervous System Diseases, the Affiliated Hospital of Xuzhou Medical University Xuzhou Medical University Xuzhou China; ^2^ Department of Neurosurgery the Affiliated Hospital of Xuzhou Medical University Xuzhou China; ^3^ Department of Neurosurgery Henan Provincial People's Hospital People’s Hospital of Zhengzhou University Zhengzhou China; ^4^ Department of Neurosurgery Kaifeng Central hospital Kaifeng China; ^5^ Blood Diseases Institute Xuzhou Medical University Xuzhou China

**Keywords:** cell proliferation, Csnk1a1, GBM, pro‐inflammatory factors, radiotherapy

## Abstract

Glioblastoma multiforme (GBM), a fatal brain tumour with no available targeted therapies, has a poor prognosis. At present, radiotherapy is one of the main methods to treat glioma, but it leads to an obvious increase in inflammatory factors in the tumour microenvironment, especially IL‐6 and CXCL1, which plays a role in tumour to resistance radiotherapy and tumorigenesis. Casein kinase 1 alpha 1 (CK1α) (encoded on chromosome 5q by Csnk1a1) is considered an attractive target for Tp53 wild‐type acute myeloid leukaemia (AML) treatment. In this study, we evaluated the anti‐tumour effect of Csnk1a1 suppression in GBM cells in vitro and in vivo. We found that down‐regulation of Csnk1a1 or inhibition by D4476, a Csnk1a1 inhibitor, reduced GBM cell proliferation efficiently in both Tp53 wild‐type and Tp53‐mutant GBM cells. On the contrary, overexpression of Csnk1a1 promoted cell proliferation and colony formation. Csnk1a1 inhibition improved the sensitivity to radiotherapy. Furthermore, down‐regulation of Csnk1a1 reduced the production and secretion of pro‐inflammatory factors. In the preclinical GBM model, treatment with D4476 significantly inhibited the increase in pro‐inflammatory factors caused by radiotherapy and improved radiotherapy sensitivity, thus inhibiting tumour growth and prolonging animal survival time. These results suggest targeting Csnk1a1 exert an anti‐tumour role as an inhibitor of inflammatory factors, providing a new strategy for the treatment of glioma.

## 1 INTRODUCTION

Glioblastoma multiforme (GBM) is the most common and aggressive primary brain tumour.^1^ Its mortality rate is high, and the median survival time is low.^2^, ^3^ The key obstacles to successful treatment include tumour recurrence and resistance to radiotherapy and chemotherapy. Therefore, there is an urgent need to identify potential therapeutic targets.

The tumour microenvironment is considered to play a key role in tumour cell invasion. Mounting evidence indicates that inflammatory factors are found in the tumour microenvironment of many cancers.^4^ Pro‐inflammatory factors have been described in glioma, and it is generally considered that IL‐6, IL‐8, CXCL1, CXCL10 and TGFβ2 are pro‐tumour cytokines closely related to glioma progression.^5^, ^6^, ^7^, ^8^, ^9^ Interleukin‐6 (IL‐6) is a cytokine that regulates immune and inflammatory reactions. It is expressed in various solid tumours and promotes the invasion and migration of tumour cells.^10^, ^11^, ^12^, ^13^ IL‐6 induces invasion and migration in glioma cells through JAK‐STAT3, MAPK and PI3K/AKT signalling pathways, and its expression is closely related to prognosis.^14^ CXCL1, like IL‐6, affects the invasion and migration of tumour cells and induces the recruitment of a variety of cytokines to form a tumour microenvironment that promotes the proliferation of tumour cells.^7^, ^8^, ^15^ The transforming growth factor (TGF)‐β/Smad pathway seems to play an important role in GBM tumorigenesis, resistance to common therapies and poor clinical outcome.^16^

In the tumour microenvironment, inflammatory factors interact with EGF, PDGF, ERK1/2 and P65 signalling pathways. The role of inflammatory factors in the tumour microenvironment is very complex and related to tumour progression and recurrence.^17^ At present, radiotherapy plays an important role in the treatment of glioma, but significantly increases CXCL1, IL‐6 and IL‐8 amounts.^18^ After acquiring the radioresistance phenotype, transcriptome analysis showed that pro‐inflammatory pathways are obviously activated, with IL‐6 as the main participant,^19^ providing the basis for targeted therapies aiming to block inflammatory pathways to enhance the sensitivity of GBM to radiotherapy.

Csnk1a1 (CK1a) is one of the seven subfamilies of casein kinase 1, which is necessary for the survival of acute myeloid leukaemia cells. This indicates that Csnk1a1 is a treatment target in acute myeloid leukaemia. It was found that knocking out Csnk1a1 reduces Rps6 phosphorylation in AML cells, induces p53 expression and inhibits tumour cell proliferation. Importantly, TP53‐null leukaemia has no response to gene knockdown, and the biological effect of Csnk1a1 inhibition depends on the expression of TP53.^20^ Therefore, Csnk1a1 inhibition may be used in TP53 wild‐type tumours. At present, Csnk1a1 has become a potential therapeutic target in many tumours, including chronic lymphocytic leukaemia,^21^ acute myeloid leukaemia ^20^ and multiple myeloma.^22^, ^23^ It has been shown that inhibition of Csnk1a1 suppresses the NF‐κB signalling pathway and reduces the resistance of EGFR mutant to erlotinib in non‐small‐cell lung cancer.^24^ In addition, Csnk1a1 activates NF‐κB signalling.^25^ NF‐κB is the main signalling pathway in inflammation ^17^ and expression changes in inflammatory factors constitute an important mechanism of glioma resistance to radiotherapy.

In this study, we evaluated the effect of Csnk1a1 inhibition on the proliferation of glioblastoma, inflammatory factors and sensitivity to radiotherapy. This preclinical study provides support for clinical trials of Csnk1a1 inhibitors in GBM treatment.

## 2 MATERIALS AND METHODS

### 2.1 Patient tissue samples

In this study, 26 patient tissues were collected, including nine non‐tumour brain tissues (patients with brain trauma) and 17 tissues (Grade II: 4, Grade III: 4, Grade IV: 9). All specimens were collected from the Affiliated Hospital of Xuzhou Medical University (Xuzhou, China). Glioma patients werehistologically diagnosed according to World Health Organization (WHO) criteria. None of the patients received any therapies before sample collection, such as radiation, immunotherapy or chemotherapy.Written informed consent was obtained from all patients. This study was approved by Research Ethics Committee of the Affiliated Hospital of Xuzhou Medical University.

### 2.2 Cell lines and reagents

Human GBM cell lines (U87, U251, A172, T98G, LN229 and LN18) used in this study were cultured and maintained in Dulbecco's modified Eagle's medium (DMEM) supplemented with 10% foetal bovine serum (FBS). GSC2 was cultured in neurobasal medium containing basic fibroblast growth factor, epidermal growth factor, B27 supplement, harpin, l‐glutamine and N2 supplement.^26^ Primary antibodies for Csnk1a1, β‐actin, P65, p‐P65, p‐IkB and IkB were purchased from Cell Signaling Technology (CST, Beverly, MA, USA). p‐FOXO1a (S322/S325) and FOXO1a were purchased from Abcam (Cambridge, MA, USA). Csnk1a1 inhibitor D4476 was purchased from Selleck Chemicals (Houston, TX, USA). D4476 was dissolved in DMSO to create a 10 mmol/L solution, which was diluted to different concentrations before use.

### 2.3 Construction of lentiviruses and establishment of stable cell lines

In the pHBLV‐U6/GFP/Puro vector, short hairpin RNA targeting the Csnk1a1 gene and the non‐targeted control sequence were constructed (Table S1). The lentivirus was produced by co‐transfection of the lentivirus vector and two packaging vectors in 293FT cells with the PolyJet™ transfection reagent. After 48 h of culture, the supernatant was collected and concentrated by ultracentrifugation. For silencing, LN229 and U87 cells were transduced with shRNA and control lentiviruses for 72 h, respectively. Puromycin at 2.5 μg/ml was used for selection. Stable cell lines were obtained by culturing viable cells.

For Csnk1a1 overexpression, the overexpression plasmid (p3×Flag‐Csnk1a1) was provided by Jiman Biotechnology Company (Genomeditech, Shanghai, China) and was transfected into LN229 and U87 cells with Lipofectamine™ 2000 reagent (Invitrogen, Carlsbad, CA, USA). After transfection for 48 h, the cells were harvested for Western blot analysis and in vitro cell viability and colony formation assays.

### 2.4 Cell viability assay

Cell counting kit‐8 (Dojindo, Kumamoto, Japan) was used to detect the cell viability. The GBM cell lines were inoculated into 96‐well plates with 3000 cells per well, and D4476 with different concentrations was added. After 72 h, 10 μL of CCK‐8 solution was added to each well. Two hours later, the optical density of 450nm (OD450) was measured with a microplate reader. In order to standardize the results, the background reading of the medium was subtracted from each well.

### 2.5 EdU incorporation assay

The cell light EdU cell proliferation detection kit (Ruibo Biotechnology, Guangzhou, China) was used for detecting cell proliferation.^27^ Cells stably expressing shRNA or transfecting with p3 × Flag‐Csnk1a1 treated with different concentrations of D4476 were cultured in 96‐well plates. After 24 h, the cells were incubated with 50 μL EdU for 4 h, fixed with 4% paraformaldehyde for 15 min and treated with 0.5% Triton X‐100 for 20 min. Cells were then incubated in the dark with 1× Apollo reaction mixture for 30 min and stained with DAPI for 20 min. After washing with PBS for three times, cell images were acquired under a fluorescence inverted microscope.

### 2.6 Colony‐forming assay

U87 and LN229 cells were seeded into 6‐well plates. There were 500 cells per well and three replicate wells per group. The experimental group was treated with D4476 at the specified concentrations (0, 5 and 10 μM), combined with radiotherapy at 4 Gy. 24 h later, further culture was performed for 10‐14 days with fresh drug‐free medium. Then, cells were washed with PBS and fixed with methanol and stained with 0.1% crystal violet solution. After washing, the cells were imaged and counted under a microscope.

### 2.7 Cell cycle and apoptosis analyses

U87 and LN229 cells were treated with D4476 at specified concentrations, and shRNA cells, in combination with radiotherapy, were cultured for 24 h. Then, the cells were centrifuged at 1000 rpm for 5 min at 4°C and fixed with 70% cold methanol overnight. After washing twice with PBS, the cells were stained with a staining solution containing 50 μg/mL propidium iodide (PI) and 25 μg/mL ribonuclease (RNase) for 30 min. Flow cytometry (Becton‐Dickinson) was used to analyse cell cycle with the integrated flow cytometry software (Becton‐Dickinson).

For apoptosis assessment, treated cells were collected, washed twice with pre‐cooled PBS and resuspended in pre‐cooled binding buffer. Next, about 5 μL Annexin V‐FITC and 5 μL PI were added to the cell suspension, for 10 min of incubation on ice in the dark. Flow cytometry was used to detect apoptosis.

### 2.8 ELISA assay

IL‐6 (PD6050), CXCL1 (DY275), and TGFβ2 (DY302) DuoSet ELISAs were purchased from R&D Systems and carried out as per manufacturer's instructions.

### 2.9 Western blot analysis

Csnk1a1 overexpression cells and LN229 cells were incubated with different concentrations of D4476 in combination with 4 Gy radiotherapy, and total protein was extracted at different times. Total protein concentration was measured by the Bradford method. Then, 50 mg of total protein underwent separation by 10% SDS‐polyacrylamide gel electrophoresis and electro‐transfer onto polyvinylidene fluoride (PVDF) membranes. Subsequently, the membranes were successively incubated with the indicated primary antibodies at 4°C and secondary antibodies for 2 h at room temperature. The signals were detected with the ECL detection system.

### 2.10 RNA extraction and qPCR

Total RNA was extracted from control, Csnk1a1 knockdown cells and mouse brain tumour tissue samples using TRIzol (Invitrogen) according to the manufacturer's protocol. Reverse transcription was performed with the Transcript First Chain cDNA Synthesis Kit (Roche). qPCR was performed as described in our previous study.^28^ The gene‐specific primers are listed in Table S2. TBP gene expression was used for normalization.

### 2.11 Neurosphere formation assay

GSC2 cells were seeded into 96‐well plates at 1000 cells per well.^26^ The cells were cultured in Neurobasal^TM^ medium containing D4476 (2 or 4 μM), and formation of colony spheres was assessed under a microscope after 10‐14 days. Neurospheres with more than 50 cells were counted in each well.

### 2.12 In vivo studies

Animal experiments were approved by the Ethics Committee of Xuzhou Medical University. In this study, 5‐ to 6‐week‐old male BALB/c athymic nude mice were purchased from Weitong Lihua Experimental Animal Technology (Beijing, China). GCS2 cells (5 × 10^5^ cells per mouse) were injected intracranially into the right striatum of these mice with a small animal stereotactic apparatus.^29^ Five days later, nude mice bearing tumour cells were randomly divided into four groups (14 mice in each group), including the control, D4476 (50 mg/kg; intraperitoneally administered every other day), radiotherapy (2 Gy every other day, total 10 Gy) and D4476 + radiotherapy (D4476 at 50 mg/kg combined with radiotherapy at 2 Gy, every other day) groups. Seven mice in each group were randomly selected and killed after 25 days of treatment. Tumours were extracted for H&E staining and mRNA extraction. The remaining seven mice in each group were used for survival analysis.

### 2.13 Statistical analysis

Each experiment was repeated independently for more than three times. SPSS 22.0 is used for statistical analyses. The data were presented as the means ± SEM of three independent experiments. The unpaired *t* test was used to calculate the difference between the control group and the treatment group. Kaplan‐Meier survival curve was used for the survival analysis. *P* values <0.05 were considered statistically significant.

## 3 RESULTS

### 3.1 High Csnk1a1 expression in high‑grade glioma tissues leads to poor prognosis in glioma patients

**FIGURE 1:**
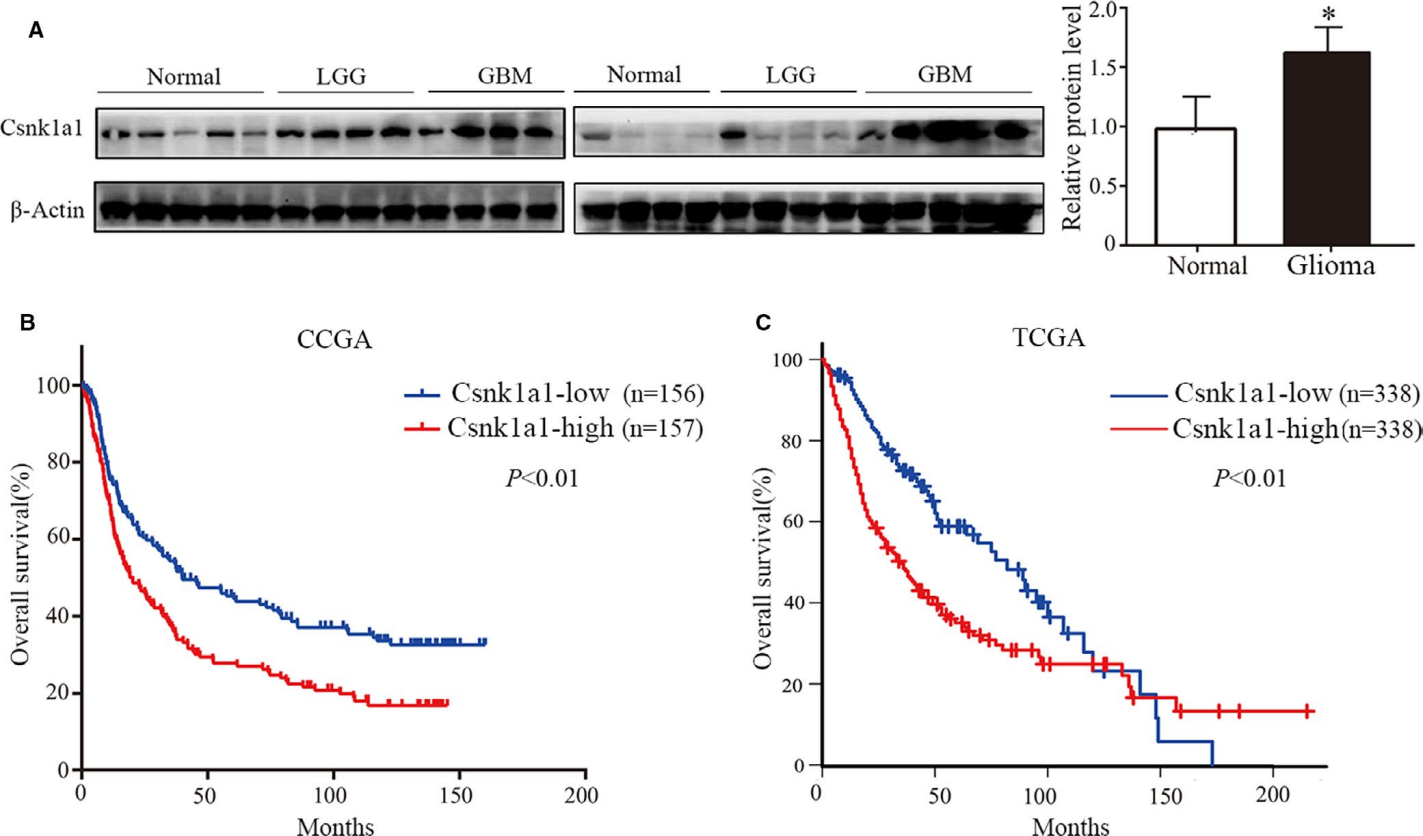
High expression of Csnk1a1 in glioblastoma patients is associated with poor prognosis. A, The expression of Csnk1a1 in non‐tumour brain tissues and glioma tissues was detected by Western blot. And statistical analysis was carried out (*P < .01). B, Kaplan‐Meyer survival analysis of 313 glioma patients using Chinese Glioma Genome Atlas, 157 tissues had high Csnk1a1 expression (*P < .01). C, Overall survival rate of 676 glioma patients in Cancer Genome Atlas glioblastoma data set was analysed by GEPIA analysis, and 338 tissues had high Csnk1a1 expression (*P < .01)

To examine the role of Csnk1a1 in the pathogenesis of glioma, firstly, Western blotting was performed to analyse the difference in Csnk1a1 protein expression between glioma and non‐tumour brain tissue samples. Compared with normal brain tissue, Csnk1a1 was highly expressed in glioma (Figure 1A). The protein expression in tumour tissue samples was about 1.5 times as high as that of non‐tumour tissue specimens. Csnk1a1 was also highly expressed in glioma tissue samples using GEPIA analysis in the TCGA database (Fig. S1A). TCGA and CCGA databases were used to further analyse the relationship between the expression of Csnk1a1 and prognosis in glioma. The prognosis of glioma patients with high expression of Csnk1a1 was worse than that of cases with low expression (Figure 1B,C), suggesting that Csnk1a1 plays an important role in the occurrence and development of glioma, and could be used as a marker of poor prognosis.

### 3.2 Csnk1a1 promotes cell proliferation and colony formation in GBM cells

**FIGURE 2:**
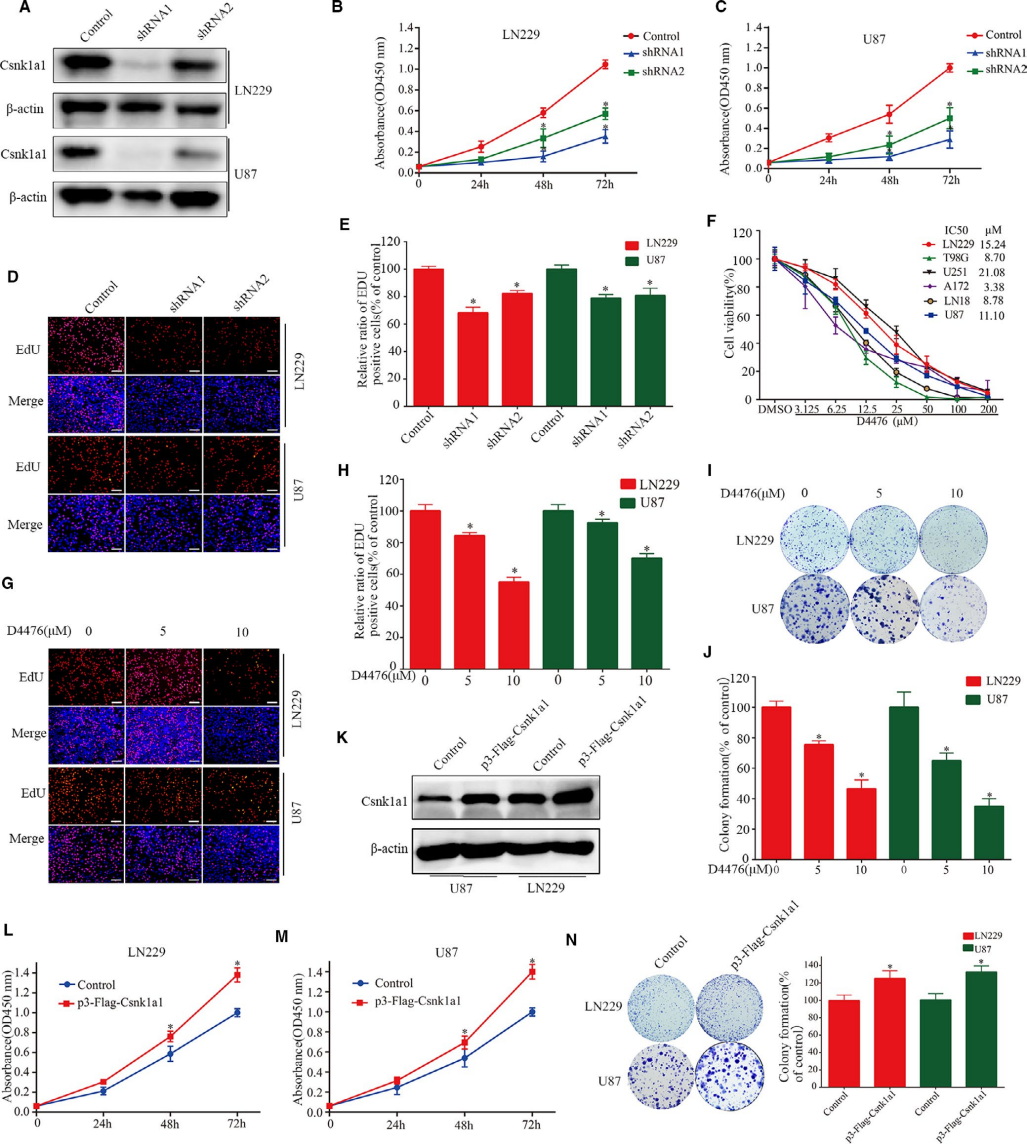
Csnk1a1 down‐regulation and inhibition suppress GBM cell proliferation and colony formation. A, Down‐regulation efficiency of Csnk1a1 silencing in LN229 and U87 cells, confirmed by immunoblotting. B and C, Viability abilities of LN229 and U87 cells after Csnk1a1 knockdown, detected by CCK‐8 assay. D and E, Anti‐proliferative effects after Csnk1a1 down‐regulation, determined by the EdU incorporation assay. Scale bar: 100 μm. F, GBM cells were treated with different concentrations of D4476 for 72 h, and cell viability was determined by CCK‐8 assay. G and H, The EdU incorporation method was used to determine the anti‐proliferative effect of D4476. The number of proliferating cells was normalized with the control group. Scale bar: 100 μm (*P < .05). I and J, LN229 and U87 cells were treated with different concentrations of D4476 for 24 h, and the numbers of colony formed were counted, relative to the control group (*P < .05). K Csnk1a1 overexpression in LN229 and U87 cells, confirmed by immunoblotting. L and M, Viability abilities of LN229 and U87 cells after Csnk1a1 overexpression, detected by CCK‐8 assay. N, Csnk1a1 overexpression enhances colony formation in U87 and LN229 cells. Quantitative analysis of the results of the colony formation experiment was performed (*P < .05). The data from three independent experiments were expressed as the means ± SEM (*P < .05)

Csnk1a1 is a multifunctional protein that inhibits the tumour suppressor Tp53 and promotes the progression of tumour cells.^22^, ^23^ TP53 wild‐type U87 and TP53 mutant‐type LN229 cells were screened out. The effect of Csnk1a1 knockdown was verified by Western blot (Figure 2A). In order to verify the effect of Csnk1a1 on GBM cell proliferation, U87 and LN229 cells were treated with shRNA. Cell proliferation was then measured by CCK‐8 and EdU assays. As shown in Figure 2B‐E, down‐regulation of Csnk1a1 significantly inhibited GBM cell survival and proliferation, with less EdU‐positive cells compared with the control group. In order to further verify the effect of Csnk1a1 on GBM cell proliferation, U87 and LN229 cells were transfected with p3‐Flag‐Csnk1a1. The effect of Csnk1a1 overexpression was examined by Western blot (Figure 2K). Cell proliferation was then measured by CCK‐8 and colony formation assays. As shown in Figure 2L‐N, overexpression of Csnk1a1 significantly enhanced GBM cell survival and proliferation, and the number of colonies cells was increased compared with the control group.

D4476 was an inhibitor of Csnk1a1, which inhibits the CK1‐mediated phosphorylation of FOXO1a (Fig. S1B).^30^ Cell counting kit‐8 (CCK‐8) was used to evaluate the effect of the Csnk1a1 inhibitor D4476 on the cell viability of six GBM cell lines. The results showed that D4476 significantly inhibited the viability of all six GBM cell lines in a dose‐dependent manner, with IC50 values ranging from 3.38 to 21.08 µM (Figure 2F). In addition, D4476 had obvious inhibitory effects on the growth of TP53 mutant‐type and TP 53 wild‐type GBM cells. U87 and LN229 cells were treated with D4476 (10 μM), and the percentages of EdU‐positive cells were decreased to 70.02% and 55.06%, respectively (Figure 2G,H). These results indicated that D4476 may be a potential target drug for GBM, regardless of TP53 expression. To observe the long‐term inhibitory effect of D4476 on GBM cell proliferation, we further evaluated the colony formation abilities of U87 and LN229 GBM cell lines after D4476 treatment. We found that D4476 significantly decreased the number of colonies (Figure 2I,J). In summary, inhibiting Csnk1a1 activity significantly suppressed survival, proliferation and colony formation in GBM cell lines. These findings suggested that Csnk1a1 plays a role in promoting glioma proliferation, and could be used as a therapeutic target.

### 3.3 Csnk1a1 induces the production of pro‐inflammatory factors

**FIGURE 3:**
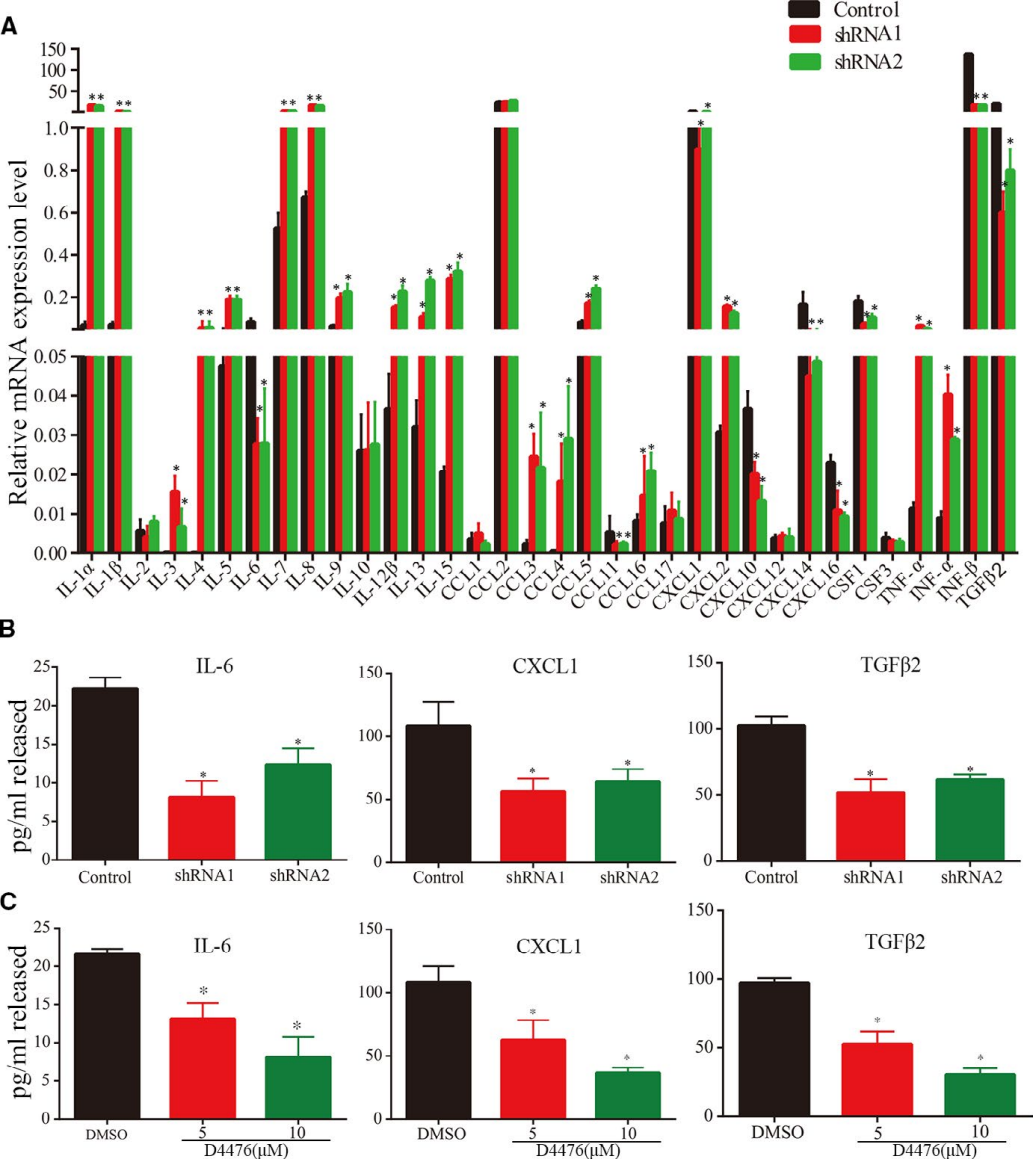
Csnk1a1 alters the production and secretion of pro‐inflammatory factors. A, LN229 cells were engineered to express shRNA or vector control. Then, mRNA levels of tumour cytokines were quantified by qPCR. B, Cytokine secretion was quantified in conditioned medium with ELISA kits specific to IL‐6, CXCL1 and TGFβ2, respectively, in cells treated with vehicle alone or shRNA (n = 3) (*P < .05). C, IL‐6, CXCL1 and TGFβ2 quantification in conditioned medium collected from LN229 cells incubated for 24 h in serum‐containing medium supplemented with vehicle alone or D4476 (n = 3). These data from three independent experiments were expressed as the means ± SEM (n = 3, *P < .05)

Csnk1a1 may exert its anti‐tumour activity by inhibiting NF‐κB signalling pathway,^25^ which is the main signalling pathway of inflammation.^17^ We hypothesized that Csnk1a1 may influence the secretion of tumour‐promoting cytokines. In order to test this hypothesis, we extracted the mRNA from Csnk1a1 knockdown cells and mRNA levels of 34 different inflammation factors were assessed by quantitative polymerase chain reaction (qPCR) (Figure 3A). These results revealed many changes in cytokine expression, but IL‐6, CXCL1 and TGFβ2 were significantly decreased, which play important roles in tumour genesis and development.

We further confirmed the secretion level changes in the three cytokines by enzyme‐linked immunosorbent assay (ELISA). Indeed, secreted IL‐6, TGFβ2 and CXCL1 amounts after Csnk1a1 knockdown were decreased (Figure 3B). Similarly, D4476 significantly inhibited the production and secretion of IL‐6, CXCL1 and TGFβ2 (Figure 3C). These results suggested Csnk1a1 affects the secretion of pro‐inflammatory cytokines, providing the basis for targeted therapies based on the blockade of inflammatory pathways.

### 3.4 Csnk1a1 inhibition enhances sensitivity to radiotherapy

**FIGURE 4:**
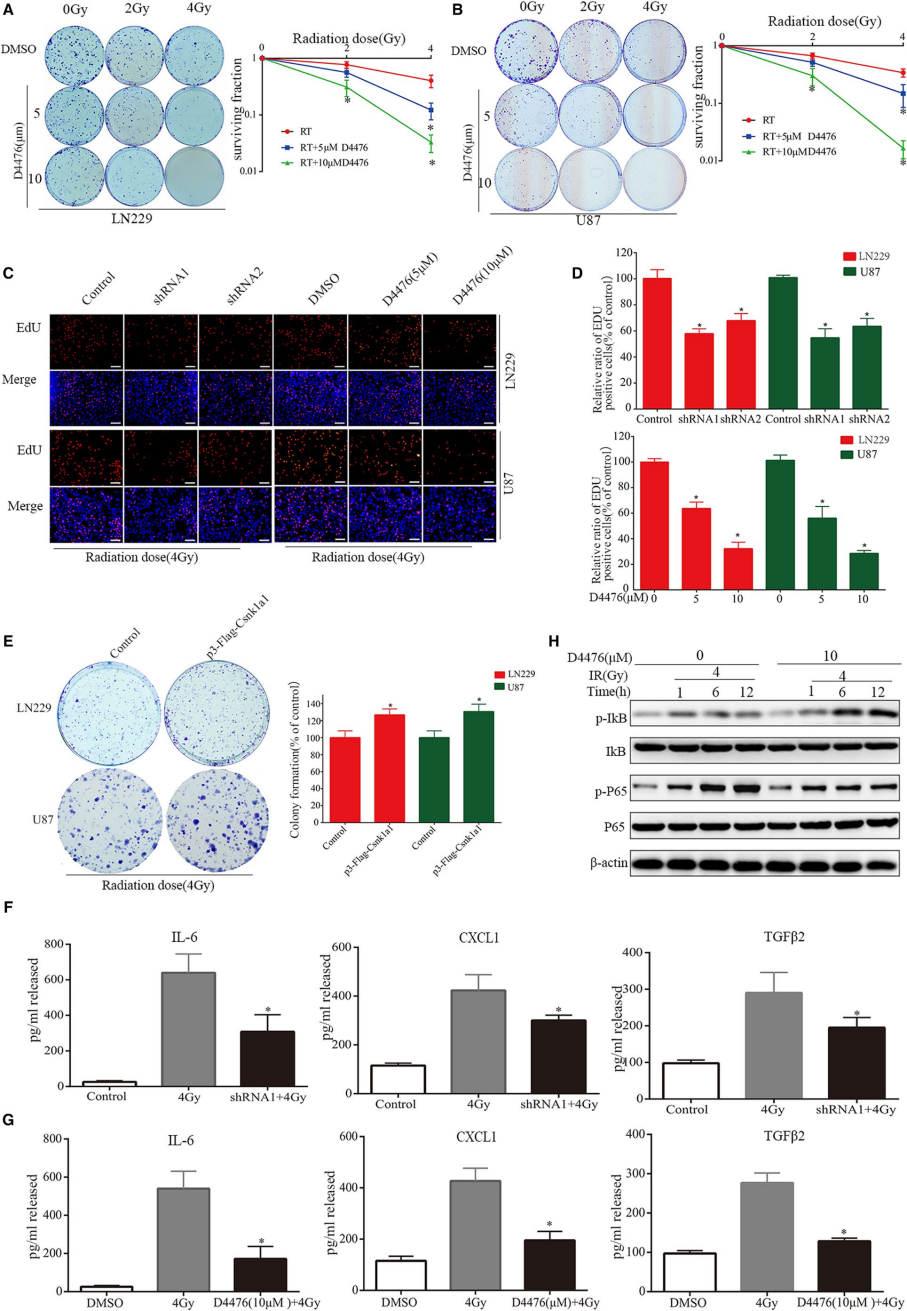
Csnk1a1 inhibition enhances sensitivity to radiotherapy. A and B, Colonies formed after administration of D4476 alone, radiotherapy alone and combined treatment, respectively, were counted in the colony formation assay. C, The effects of shRNA and D4476, radiation alone or their combination on cell proliferation were assessed by the EdU incorporation test. Scale bar: 100 μm. D, Quantitative analysis of proliferative cell numbers. The numbers of proliferative cells were normalized to that of the control group (*P < .05). E, Csnk1a1 overexpression enhances colony formation in U87 and LN229 cells in combination with radiotherapy. Quantitative analysis of the results of the colony formation experiment was performed (*P < .05). F and G, Secreted IL‐6, CXCL1 and TGFβ2 amounts in LN229 cells treated with shRNA or D4476 combined with radiotherapy were determined by ELISA. Compared with radiotherapy alone, it has statistical significance (*P < .05). H, Western blotting was performed to confirm the changes in p‐P65, P65, p‐IkB and IkB in the control and D4476 combined with radiotherapy groups, β‐actin serving as a loading control. All the data are presented as means ± SEM. *P < .05

Above results showed that inhibition of Csnk1a1 activity significantly reduced the expression and secretion of IL‐6, TGFβ2 and CXCL1. Previous studies have suggested that chemokines and their receptors play a vital role in the interaction between tumour microenvironment and cancer cells, often leading to a failure of therapy.^31^ In GBM, after obtaining the radioresistance phenotype, transcriptome analysis showed that pro‐inflammatory pathways were obviously activated.^19^ Therefore, we hypothesized that inhibiting Csnk1a1 activity could promote radiotherapy sensitivity in glioma. In U87 and LN229 GBM cells, D4476 combined with radiotherapy significantly reduced colony formation (Figure 4A,B). Then, EdU was used to analyse the effect of Csnk1a1 inhibition combined with radiotherapy on cell proliferation. The number of EdU‐positive cells in the combined treatment group was significantly lower than those of the radiotherapy group (Figure 4C,D). On the contrary, compared with radiotherapy alone, Csnk1a1 overexpression significantly increased the number of colonies and EdU‐positive cells under radiotherapy (Figure 4E and Fig. S1C‐D). Therefore, Csnk1a1 plays an important role in radiotherapy resistance.

We further evaluated the changes in IL‐6, TGFβ2 and CXCL1 secretion after combined treatment with irradiation and Csnk1a1 inhibition. After irradiation, the secretion of IL‐6, TGFβ2 and CXCL1 increased significantly, but decreased significantly after combination with Csnk1a1 down‐regulation (Figure 4F). The same results were obtained with D4476 treatment with irradiation (Figure 4J). We further examined the effect of D4476 treatment on NF‐κB signalling pathway. We found that radiotherapy‐activated NF‐κB signalling pathway was obviously inhibited by D4476 treatment. D4476 treatment blocked the radiation‐induced phosphorylation of p65, and increased the levels of p‐IκB (Figure 4H). These findings indicated that Csnk1a1 may regulate the secretion of cytokines by inhibiting the NF‐κB signalling pathway.

### 3.5 Csnk1a1 significantly induces cell cycle arrest and apoptosis in combination with radiotherapy

**FIGURE 5:**
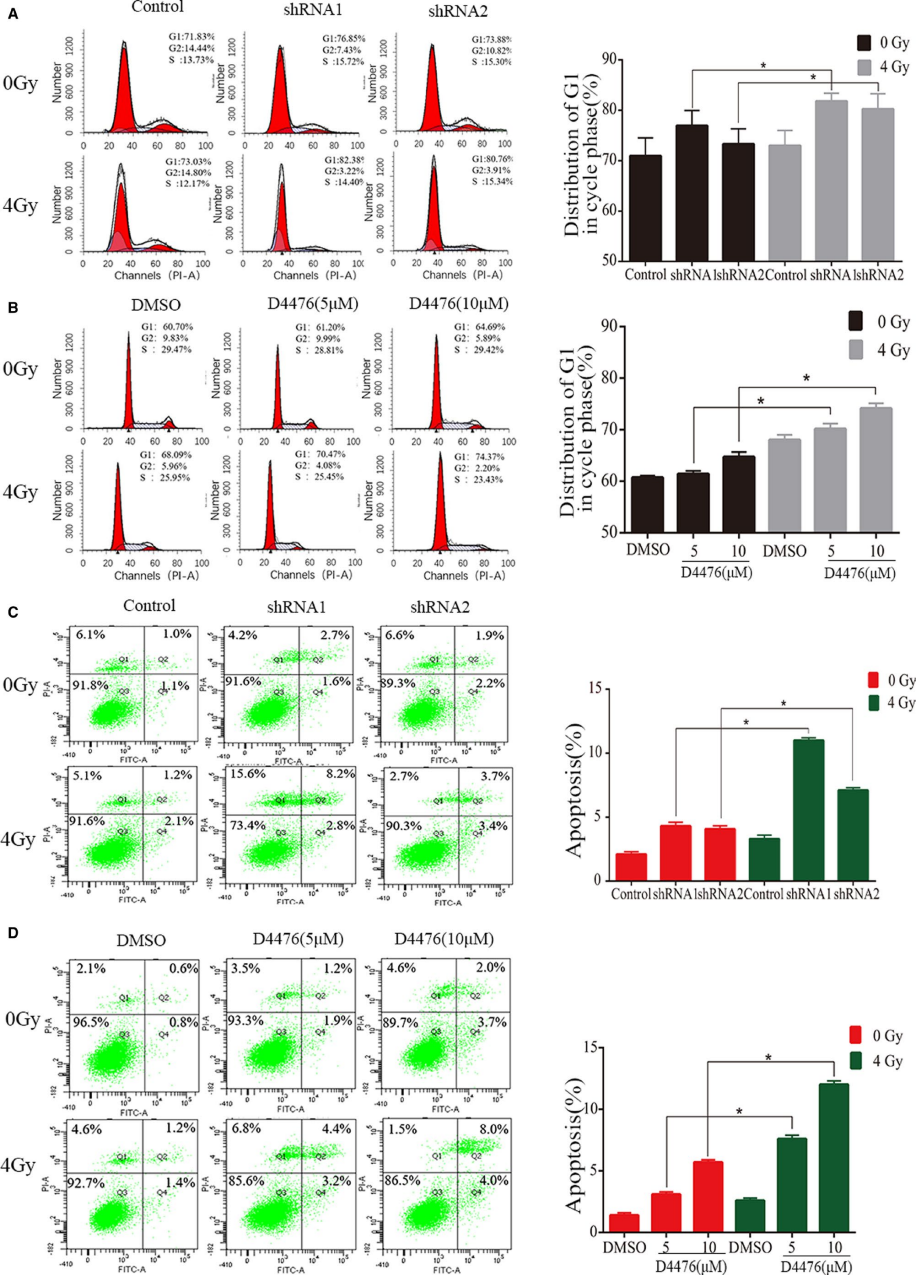
Csnk1a1 significantly induces cell cycle arrest and apoptosis in combination with radiotherapy. A and B, Representative data of cell cycle analysis of cells treated with D4476 combined with radiotherapy. LN229 cells were treated with shRNA and D4476, in combination with radiotherapy, for 24 h at the specified concentrations. Cell cycle distribution was evaluated by flow cytometry. Quantitative analysis of cell cycle distribution. Data from three independent experiments are expressed as means ± SEM. *P < .05. C and D, LN229 cells were treated with shRNA and D4476, in combination with radiotherapy, for 24 h at the specified concentrations. Apoptosis rates were evaluated by Annexin V/PI staining. All the data are presented as means ± SEM. *P < .05

We further evaluated the effect of combined therapy on GBM cell cycle and apoptosis. The number of cells in the G1‐phase was significantly increased after Csnk1a1 knockdown combined with radiotherapy (Figure 5A). Meanwhile, the Csnk1a1 inhibitor D4476 also blocked cells at the G1‐phase (Figure 5B). Furthermore, the effect of Csnk1a1 knockdown combined with radiotherapy on apoptosis was detected by flow cytometry. After combined treatment, the apoptosis rate was 13% (Figure 5C). Compared with the radiotherapy and shRNA alone groups, the apoptosis rate was increased significantly in the combination group. Meanwhile, after combined treatment with D4476, the apoptosis rate was 12% (Figure 5D). Compared with the radiotherapy and D4476 alone group, the apoptosis rate was increased significantly in the combination group. Therefore, inhibiting the activity of Csnk1a1 significantly improved cell sensitivity to radiation therapy.

### 3.6 D4476 combined with radiotherapy inhibits the growth of GBM in vivo and enhances survival time of tumour bearing mice

**FIGURE 6:**
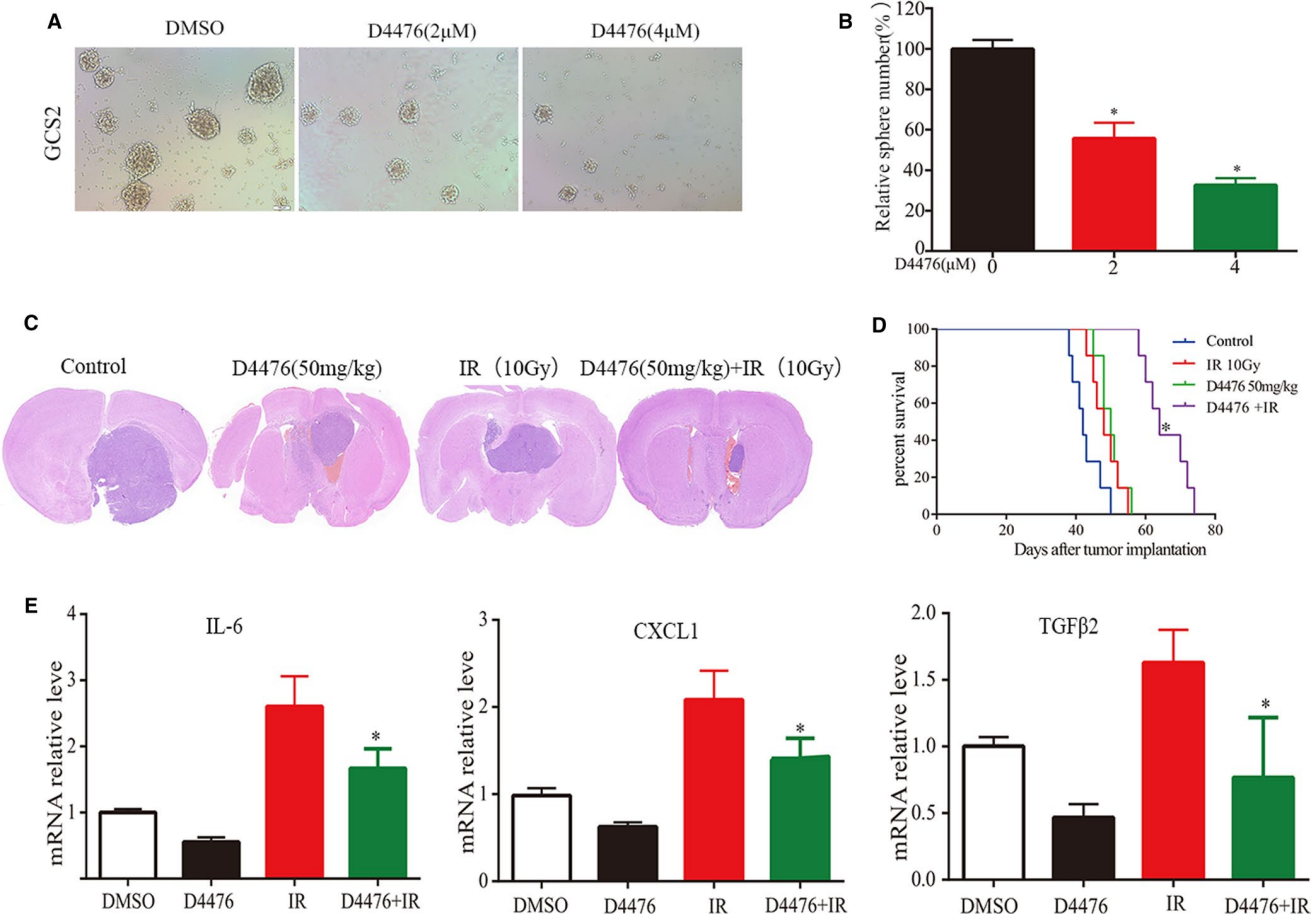
D4476 inhibits GBM growth in an intracranial xenograft mouse model and enhances tumour sensitivity to radiotherapy. A and B, Effects of D4476 on GSC2 neurosphere formation. Representative images of GSC2 neurosphere and the quantification of sphere numbers of GSC2 treated with indicated doses of D4476 or DMSO. C, The xenograft model was established by injecting 5 × 10^6^ GCS2 cells into the right striatum of the brain of nude mice. Intravenous injection of D4476 at 50 mg/kg was performed every other day. Radiotherapy, alone or in combination with D4476 treatment, was administered every other day at 2 Gy. After 4 weeks of treatment, mice were killed. Representative images of H&E staining of brain coronal sections of mice with tumours in situ. D, Kaplan‐Meier diagram showing the survival rate of animals treated with D4476 combined with radiotherapy compared with that of animals administered D4476 or radiotherapy alone (n = 7, *P < .05). E, The quantification of IL‐6, CXCL1 and TGFβ2 mRNA levels in xenograft samples from D4476 + radiotherapy groups compared with that of animals administered D4476 or radiotherapy alone (n = 3), determined by qPCR. All the data are presented as means ± SEM. *P < .05

In order to further determine the therapeutic effect of D4476 in vivo in a more clinically relevant model of GBM, we constructed a glioma stem cell (GSC)‐driven xenograft tumour mouse model. Firstly, we confirmed D4476 treatment inhibited GSC2 neurosphere formation in vitro (Figure 6A,B). Then, GCS2 cells were injected into the right hemisphere of nude mice,^26^ and the sizes of the GBM xenografts were examined, as well as the survival of GBM‐bearing mice. The animals were randomly divided into the control, 50 mg/kg D4476 alone, radiotherapy alone and combination (radiotherapy combined with D4476 treatment) groups (Table S3). The tumour size in treatment with D4476 was smaller than those in the control group. Therefore, D4476‐treated mice showed increased survival rate. Significantly, D4476 treatment combined with radiotherapy obviously inhibited the growth of tumour (Figure 6C). The survival time of mice bearing GBM was significantly increased in the combination group (Figure 6D). Compared with the control group, the median survival time of mice in the D4476 treatment group was extended by 8 days; radiotherapy treatment extended survival by 6 days, and D4476 combined with radiotherapy resulted in a 22‐day extension (Table S3).

We further analysed the expression level of inflammatory factors in tumour tissues. The results showed that the mRNA expression levels of inflammatory factors were increased significantly after radiotherapy, but decreased obviously after combination with D4476 (Figure 6E), which is consistent with the results of in vitro experiments. Taken together, radiotherapy combined with D4476 significantly inhibited tumour growth, decreased the expression of inflammatory factors and enhanced the curative effect of radiotherapy.

## 4 DISCUSSION

Targeted therapy with Csnk1a1 has achieved remarkable results, with successful treatment in ALM and lung tumours.^20^, ^24^ Casein kinases are a group of highly conserved serine‐threonine kinases that modify substrates implicated in several signalling pathways, including Wnt, Ras, NF‐kB and p53 pathways.^23^ Csnk1a1, a negative regulator of the Wnt signalling pathway, functions as an important tumour suppressor.^32^ Its ablation, combined with p53 deficiency, gives rise to tumorigenic transformation and a highly invasive phenotype in colorectal carcinoma.^22^, ^33^ Indeed, Csnk1a1 has multiple biological functions, although its therapeutic effect on GBM is uncertain. Here, we reported that Csnk1a1 was significantly overexpressed in glioma, indicating an important role in the growth of glioma cells. In this study, we showed that the Csnk1a1 inhibitor D4476 obviously inhibited GBM cells, regardless of TP53 mutation status (TP53 mutant types, U251, LN229, T98G, LN18 cells and TP53 wild‐type, U87 and A172 cells). D4476, an inhibitor of the intracranial GBM model, prolonged mouse survival. Therefore, it may be a suitable potential targeted drug for the treatment of GBM.

Previous reports have related that Csnk1a1 signalling pathway plays an important role in tumour progression, including regulating Wnt, NF‐κB, AKT and P53 signalling pathways.^20^, ^24^, ^34^, ^35^ Re‐expression of Csnk1a1 partially rescued primary effusion lymphoma cell lines from immunomodulatory drug‐mediated toxicity.^36^ In this study, we demonstrated that Csnk1a1 is an important regulator of secreted inflammatory factors. By qPCR analyses, we showed that IL‐6, CXCL1 and TGFβ2 were significantly decreased among many cytokines. It was previously reported Csnk1a1 down‐regulation induces a senescence‐associated inflammatory response with growth arrest in colorectal cancer, which loses its growth control capacity in the absence of P53 and accelerates growth and invasiveness.^33^ However, there is no relevant report about Csnk1a1 promoting the expression of CXCL1, IL‐6 or TGFβ2. IL‐6 obviously promotes growth and radiation resistance in GBM cells.^37^ The increase in IL‐6 induced by radiation may be related to tumour regrowth. Therefore, clinical treatment with IL‐6 inhibitors may be a potential treatment strategy to induce NSCLC sensitivity to radiation.^38^ CXCL1 is highly expressed in GBM, which is positively correlated with poor patient prognosis. In addition, CXCL1 regulates the NF‐κB signalling pathway and induces the radioresistance of GBM.^39^ Increased expression of TGFβ is related to the malignant degree of glioma. TGFβ may contribute to tumour pathogenesis in many ways. TGFβ2 stimulates the expression of vascular endothelial growth factor and some metalloproteinases, and participates in vascular remodelling and angiogenesis. Inhibitors of TGFβ signal transduction can reduce the viability and invasion of glioma in animal models and are expected to constitute a new and potential anti‐tumour therapy.^6^

Long‐term inflammation is the key driving factor of carcinogenesis as it provides an ideal microenvironment for the development and growth of tumour cells. IL‐6, CXCL1 and TGFβ2 are known pro‐tumorigenic factors associated with cancer progression. The expression and secretion of IL‐6, CXCL1 and TGFβ 2 were obviously reduced by Csnk1a1 inhibition. Therefore, inhibiting Csnk1a1 activity is an effective method for suppressing the growth of tumour cells. Meanwhile, IL‐6 and CXCL1 were obviously increased in the course of glioma radiotherapy, and the inflammatory activity was obviously enhanced by transcriptome analysis of radiotherapy resistance. IL‐6, CXCL1 and TGFβ2 play an important role in glioma radiotherapy resistance. In this study, the Csnk1a1 inhibitor D4476, combined with radiotherapy, significantly prolonged the survival of mice compared with D4476 or radiotherapy alone. Therefore, D4476 may be a suitable potential targeted drug for GBM, because it inhibited the secretion of pro‐inflammatory factors and enhanced cell sensitivity to radiotherapy. Furthermore, we found that radiotherapy induced the NF‐κB signalling pathway, which was also suppressed by Csnk1a1 inhibition. We speculate that the effect of Csnk1a1 on inflammatory factors may be regulated by the NF‐κB signalling pathway.

In summary, we focused on important chemokine networks in the regulation of pro‐ and anti‐tumour mechanisms, highlighting potential therapeutic advantages of modulating these pathways in malignant glioma and other cancers.^40^ D4476 could play an anti‐tumour role as an inhibitor of inflammatory factors, providing a new target for the treatment of glioma. However, further research strategies for targeting Csnk1a1 to treat glioblastoma should address the roles of TP 53 and NF‐κB signalling pathways in the regulation of inflammatory cytokine secretion by glioma cells.

## 5 ETHICAL APPROVAL AND CONSENT TO PARTICIPATE

All animal experimental protocols were approved by the Ethics Committee of the Xuzhou Medical University.

## CONFLICT OF INTEREST

The authors declare that they have no competing interests.

## AUTHOR CONTRIBUTION

**Guanzheng Liu:** Data curation (equal); Methodology (equal); Writing‐original draft (equal). **Huan Li:** Methodology (equal); Software (equal). **Wanhong Zhang:** Methodology (equal). **Jiefeng Yu:** Data curation (equal). **Runqiu Wu:** Methodology (equal). **Xu Zhang:** Methodology (equal). **Mingshan Niu:** Data curation (equal); Software (equal). **Xuejiao Liu:** Conceptualization (equal); Funding acquisition (equal); Project administration (equal); Writing‐review & editing (equal). **Rutong Yu:** Conceptualization (equal); Investigation (equal); Project administration (equal); Resources (equal); Supervision (equal).

## Supporting information

Fig S1

Table S1

Table S2

Table S3

## Data Availability

All data sets supporting the conclusions contained in the present report are included in the manuscript.
