# Corrigendum

**DOI:** 10.1111/jcmm.17317

**Published:** 2022-05-07

In Guanzheng Liu et al.,^1^ the published article contains an error in Figure 2G. The incorrect images of EdU are used in the original publication. The corrected Figure 2 is shown below. The authors confirm that all the results and conclusions of this article remain unchanged.

**FIGURE 2:**
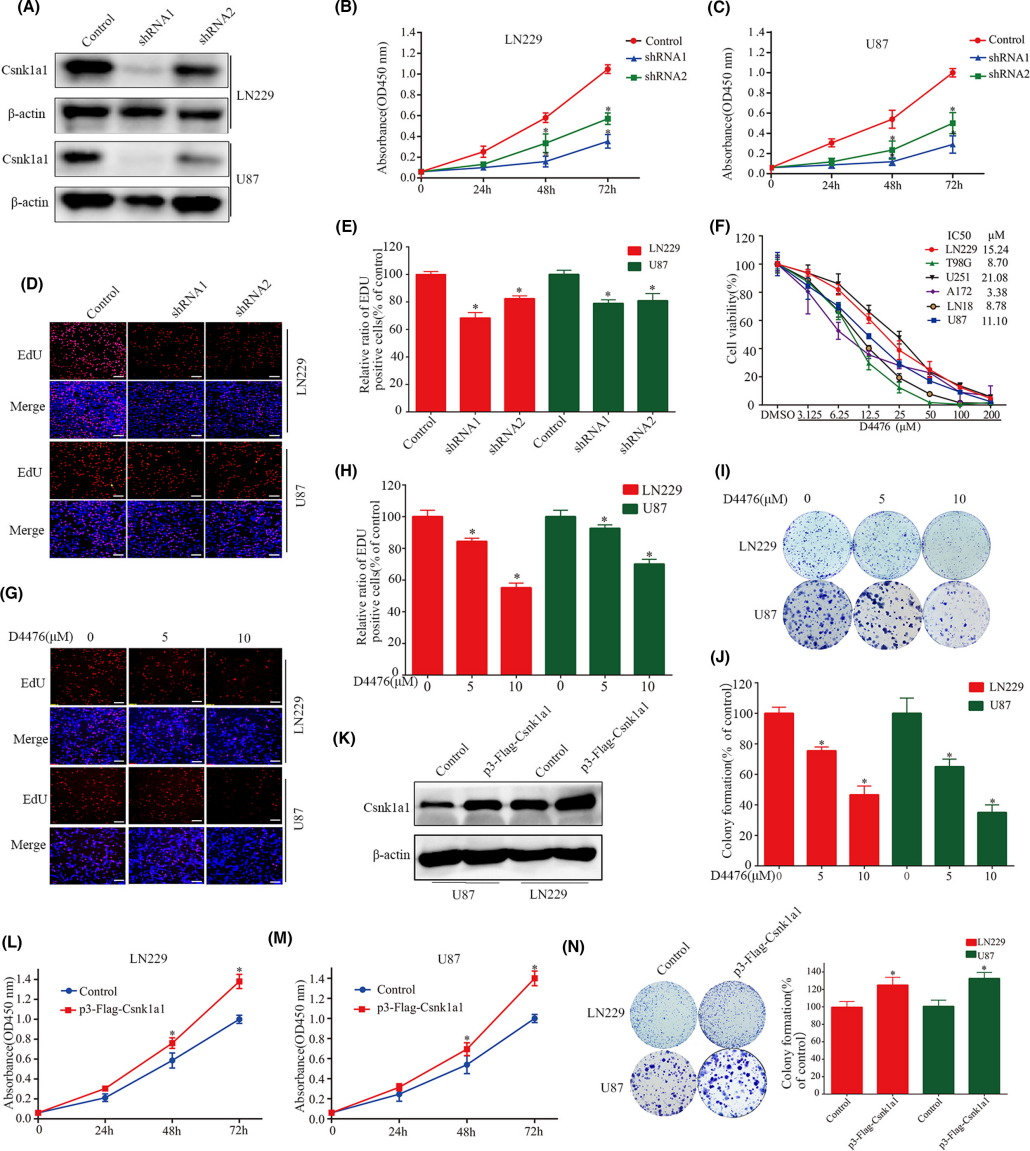
Csnk1a1 downregulation and inhibition suppress GBM cell proliferation and colony formation. (A) Downregulation efficiency of Csnk1a1 silencing in LN229 and U87 cells, confirmed by immunoblotting. (B, C) Viability abilities of LN229 and U87 cells after Csnk1a1 knockdown, detected by CCK‐8 assay. (D, E) Anti‐proliferative effects after Csnk1a1 downregulation, determined by the EdU incorporation assay. Scale bar: 100 μm. (F) GBM cells were treated with different concentrations of D4476 for 72 h, and cell viability was determined by CCK8 assay. (G, H) The EdU incorporation assay was used to determine the anti‐proliferative effect of D4476. The number of proliferating cells was normalized with the control group. Scale bar: 100 μm (*p < 0.05). (I, J) LN229 and U87 cells were treated with different concentrations of D4476 for 24 h, and the numbers of colony formed were counted, relative to the control group (*p < 0.05). (K) Csnk1a1 overexpression in LN229 and U87 cells, confirmed by immunoblotting. (L‐M) Viability abilities of LN229 and U87 cells after Csnk1a1 overexpression, detected by CCK‐8 assay. (N) Csnk1a1 overexpression enhances colony formation in U87 and LN229 cells. Quantitative analysis of the results of the colony formation experiment was performed (*p < 0.05). The data from three independent experiments were expressed as the means ±SEM (*p < 0.05)
