# Supplementary material for: Csnk1a1 inhibition modulates the inflammatory secretome and enhances response to radiotherapy in glioma
Source: J Cell Mol Med. 2021 Jul 3;25(15):7395–406. doi: 10.1111/jcmm.16767 (PMC8335695; doi:10.1111/jcmm.16767)
Supplement: Supplementary file 1 — Fig S1 [file JCMM-25-7395-s002.docx]

**Supplemental Fig**


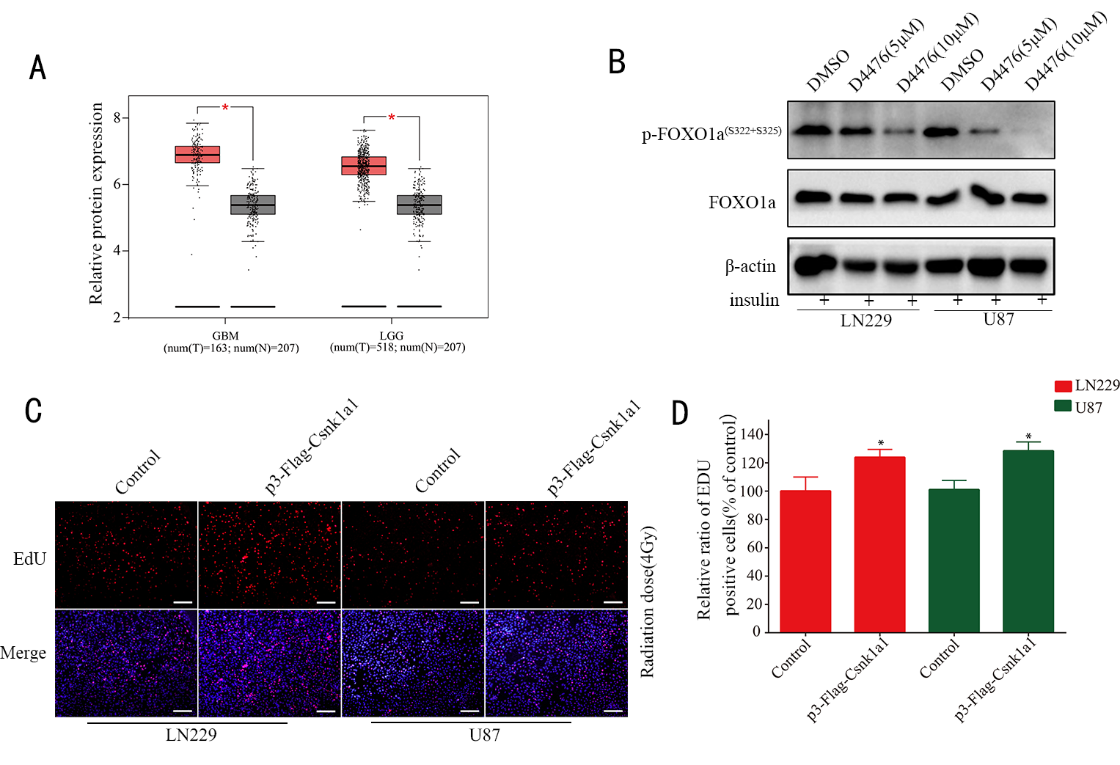


**Fig.S1 A** The relative expression of Csnk1a1 in non-tumor brain tissues and glioma tissues was analyzed by GEPIA analysis in Cancer Genome Atlas glioblastoma dataset and GTEx database. **B** LN229 and U87 cells were serum starved for 4 h, and then stimulated for 30 min with 20 nM insulin, the cells were incubated for 6 h with specified concentrations of D4476 prior to stimulation with insulin. Then D4476 suppresses the phosphorylation of FOXO1a, confirmed by immunoblotting. **C** Measurement of proliferation effects of Csnk1a1 overexpression against radiotherapy was measured by EdU incorporation test. **D** Quantitative results of EdU incorporation assay. The numbers of proliferative cells were normalized to that of the control group. All the data were presented as means ± SEM from three independent experiments (**P*<0.05).
