# Supplementary material for: Csnk1a1 inhibition modulates the inflammatory secretome and enhances response to radiotherapy in glioma
Source: J Cell Mol Med. 2021 Jul 3;25(15):7395–406. doi: 10.1111/jcmm.16767 (PMC8335695; doi:10.1111/jcmm.16767)
Supplement: Supplementary file 2 — Table S1 [file JCMM-25-7395-s003.docx]

**Supplemental Tab 1：**The sequences of shRNA-Csnk1a1 used are as follows from 5’-3’:

shRNA1-top: CCGGCATCTATTTGGCGATCAACATCTCGAGATGTTGATCGCCAAATAGATGTTTTT

shRNA1-bottom:

AATTAAAAACATCTATTTGGCGATCAACATGCTATGAATATTAACATGTTAATCGCCAAATAGATG

shRNA2-top: CCGGGCAAGCTCTATAAGATTCTTCCTCGAGGAAGAATCTTATAGAGCTTGCTTTTT

shRNA2-bottom:

AATTAAAAACTGCCTGCTTAATTGTGCTAGGCTATGAATATTAACCTAACACAATTAAACAGGCAG

shNC-top: CCGGGGATAATGGTGATTGAGATGGCTCGAGCCACTCAATCACCATTATCCTTTTT

shNC-bottom:

AATTAAAAAGGATAATGGTGATTGAGATGGCTCGAGCCACTCAATCACCATTATCC.
