# Supplementary material for: Csnk1a1 inhibition modulates the inflammatory secretome and enhances response to radiotherapy in glioma
Source: J Cell Mol Med. 2021 Jul 3;25(15):7395–406. doi: 10.1111/jcmm.16767 (PMC8335695; doi:10.1111/jcmm.16767)
Supplement: Supplementary file 3 — Table S2 [file JCMM-25-7395-s004.docx]

**Supplemental Tab 2：**The sequences of primer used are as follows from 5’-3’:

| Primer(forward) | Sequences (5′–3′) |
| --- | --- |
| IL-1α | CTGAAGAAGAGACGGTTGA |
| IL-1β | ATCTCCGACCACCACTAC |
| IL-2 | GCACCTACTTCAAGTTCTAC |
| IL-3 | TCAACAGGGCTGTCAAGAGTT |
| IL-4 | CGGCAACTTTGTCCACGGA |
| IL-5 | GAATAGGCACACTGGAGAG |
| IL-6 | TCGGTCCAGTTGCCTTCT |
| IL-7 | TTCCTCCCCTGATCCTTGTTC |
| IL-8 | GCAGCCTTCCTGATTTCT |
| IL-9 | CTCTGTTTGGGCATTCCCTCT |
| IL-10 | CAACCTGCCTAACATGCT |
| IL-12β | ACCCTGACCATCCAAGTCAAA |
| IL-13 | CCTCATGGCGCTTTTGTTGAC |
| IL-15 | ACTAACCTTCCTCCATACCA |
| CCL1 | CTCATTTGCGGAGCAAGAGAT |
| CCL2 | TAGCAGCCACCTTCATTC |
| CCL3 | AGTTCTCTGCATCACTTGCTG |
| CCL4 | TGTATGACCTGGAACTGAAC |
| CCL5 | CCAGCAGTCGTCTTTGTCAC |
| CCL16 | ACAGAAAGGCCCTCAACTGTC |
| CXCL1 | AATCCAACTGACCAGAAGG |
| CXCL2 | GGCAGAAAGCTTGTCTCAACCC |
| CXCL10 | GCTATGTTCTTAGTGGATGTTC |
| CXCL12 | ATTCTCAACACTCCAAACTGTGC |
| CXCL14 | GCGAGGAGAAGATGGTTAT |
| CXCL16 | GGCACCTGACTCTAATACC |
| CCL17 | AGACATCTGAGGACTGCT |
| CCL11 | CCCCTTCAGCGACTAGAGAG |
| CSF1 | CCCCAAAGCCATCCCTA |
| CSF3 | GCTGCTTGAGCCAACTCCATA |
| TNFα | CCTCTCTCTAATCAGCCCTCTG |
| INFα | TCTGTTCTCTAGGCTGTGA |
| INFβ | CACCACCTCCTGTTGAAC |
| TGFβ2 | TTCTACTTAATAGCCACTCGTC |
| TBP | GAGCTGTGATGTGAAGTTTCC |
