# Supplementary material for: Csnk1a1 inhibition modulates the inflammatory secretome and enhances response to radiotherapy in glioma
Source: J Cell Mol Med. 2021 Jul 3;25(15):7395–406. doi: 10.1111/jcmm.16767 (PMC8335695; doi:10.1111/jcmm.16767)
Supplement: Supplementary file 4 — Table S3 [file JCMM-25-7395-s001.docx]

**Supplemental Table 3: In vivo study, the animal experimental procedure was depicted.**

| Time |  | | Experimental procedure | | | |
| --- | --- | --- | --- | --- | --- | --- |
| 1th day |  | | We cultivate neural stem cells GSC2 (5🞩10^5^ cells per mouse) were injected intracranially into the right striatum of these mice with a small animal stereotactic apparatus | | | |
|  |  | | Control group（n=14） | D4476 group（n=14） | IR group（n=14） | D4476+IR group（n=14） |
| 6th day |  | | DMSO | 50 mg/kg | 2Gy | 50 mg/kg+2Gy |
| 8th day |  | | DMSO | 50 mg/kg | 2Gy | 50 mg/kg+2Gy |
| 10th day |  | | DMSO | 50 mg/kg | 2Gy | 50 mg/kg+2Gy |
| 12th day |  | | DMSO | 50 mg/kg | 2Gy | 50 mg/kg+2Gy |
| 14th day |  | | DMSO | 50 mg/kg | 2Gy | 50 mg/kg+2Gy |
| 30th day |  | | sacrifice(n=7) | sacrifice(n=7) | sacrifice(n=7) | sacrifice(n=7) |
|  | | survival analysis(n=7) | | | | |
| days |  | | Control group（n=14） | D4476 group（n=14） | IR group（n=14） | D4476+IR group（n=14） |
| 38 |  | | 1 |  |  |  |
| 39 |  | | 1 |  |  |  |
| 41 |  | | 1 |  |  |  |
| 42 |  | | 1 |  |  |  |
| 43 |  | | 1 |  |  |  |
| 47 |  | | 1 |  |  |  |
| 48 |  | | 1 |  |  |  |
| 45 |  | |  | 1 |  |  |
| 48 |  | |  | 1 |  |  |
| 48 |  | |  | 1 |  |  |
| 50 |  | |  | 1 |  |  |
| 51 |  | |  | 1 |  |  |
| 52 |  | |  | 1 |  |  |
| 56 |  | |  | 1 |  |  |
| 43 |  | |  |  | 1 |  |
| 45 |  | |  |  | 1 |  |
| 46 |  | |  |  | 1 |  |
| 48 |  | |  |  | 1 |  |
| 50 |  | |  |  | 1 |  |
| 52 |  | |  |  | 1 |  |
| 55 |  | |  |  | 1 |  |
| 58 |  | |  |  |  | 1 |
| 60 |  | |  |  |  | 1 |
| 62 |  | |  |  |  | 1 |
| 64 |  | |  |  |  | 1 |
| 70 |  | |  |  |  | 1 |
| 72 |  | |  |  |  | 1 |
| 74 |  | |  |  |  | 1 |

Days: The died days of the nude mice after tumor implantation

1 represents the number of died mouse

D4476 Dose (50mg/kg in 1% DMSO in PBS per mouse)

All the mice were treated with D4476 or vehicle control by intraperitoneal injection
